# National trends in neonatal and under-five mortality in Saudi Arabia (2018–2023): a descriptive ecological analysis using WHO Global Health Observatory data

**DOI:** 10.3389/fpubh.2026.1903279

**Published:** 2026-08-28

**Authors:** Eman M. Alanazi, Yara Alkhalid, Ahmed Alqheedan, Saleh Alzughaibi

**Affiliations:** 1 Department of Health Informatics, College of Health Sciences, Saudi Electronic University, Riyadh, Saudi Arabia; 2 Department of Public Health, College of Health Sciences, Saudi Electronic University, Riyadh, Saudi Arabia

**Keywords:** child health, ecological analysis, neonatal mortality, Saudi Arabia, under-five mortality, vision 2030, WHO Global Health Observatory

## Abstract

**Background:**

Neonatal mortality rate (NMR) and under-five mortality rate (U5MR) are core child-survival indicators and are influenced by, though not a direct measure of, the quality of maternal and newborn care. Saudi Arabia has achieved substantial reductions in both indicators over recent decades; however, year-to-year dynamics during the period of Vision 2030 health reforms remain incompletely described using standardized, internationally comparable data.

**Objective:**

To describe annual trends in NMR and U5MR in Saudi Arabia from 2018 to 2023 using WHO Global Health Observatory (WHO-GHO) national estimates, and to situate these descriptive trends within the broader regional and international literature on child mortality.

**Methods:**

A descriptive ecological analysis was conducted using all available national annual estimates (2018–2023; *n* = 6 per indicator) from WHO-GHO. Descriptive statistics and annual percentage change (APC) were calculated as the primary analytical approach. An unadjusted linear regression of rate on year is reported to characterize the direction and approximate magnitude of change over the period; because six annual observations provide very limited statistical power, regression *p*-values are reported for completeness only and are not used to support claims of trend presence or absence.

**Results:**

NMR declined from 3.6 to 3.0 per 1,000 live births between 2018 and 2023 (mean 3.27; range 3.0–3.6), a change concentrated mainly in 2020. U5MR fluctuated within a narrow band, falling from 6.1 in 2018 to 5.6 in 2021, rising to 6.4 in 2022, and partially returning to 6.2 in 2023 (mean 5.98; range 5.6–6.4). The unadjusted linear slopes were −0.12/year for NMR and +0.03/year for U5MR.

**Conclusion:**

Between 2018 and 2023, Saudi Arabia’s NMR and U5MR remained low and comparatively stable by international standards, with a gradual decline in NMR and a transient fluctuation in U5MR centered on 2022. These are descriptive, population-level patterns; the dataset does not include measures of healthcare quality, patient safety, or COVID-19 service disruption, and any interpretation connecting the observed trends to these factors should be regarded as a hypothesis for future research rather than a finding of this study. Future work linking subnational data, cause-specific mortality, and direct quality-of-care indicators to these trends is needed.

## 1 Introduction

The neonatal mortality rate (NMR, deaths within the first 28 days of life per 1,000 live births) and the under-five mortality rate (U5MR, deaths before age five per 1,000 live births) are widely used indicators of population health and child survival (1, 2, 16). Both indicators are influenced by a broad set of determinants that extend well beyond the health system itself, including maternal education and nutrition, fertility patterns, socioeconomic inequality, congenital and genetic disorders, environmental exposures, and national health expenditure, in addition to the availability and quality of perinatal and early childhood care (3, 4). Attributing changes in either indicator to any single determinant—including healthcare quality—therefore requires caution unless the relevant determinants are directly measured.

Globally, neonatal deaths now account for a growing share of all under-five deaths, a pattern that has prompted increasing attention to the quality, rather than only the coverage, of perinatal care in settings that have already achieved broad access to basic services (5, 6). Conditions such as prematurity, birth asphyxia, neonatal infections, and congenital anomalies dominate the neonatal disease burden (7, 15).

Saudi Arabia has recorded a substantial long-term decline in child mortality, from rates exceeding 100 per 1,000 live births in the early 1970s to single-digit NMR and U5MR today, and has met the SDG 3.2 targets (NMR ≤ 12, U5MR ≤ 25 per 1,000 live births) well ahead of the 2030 target date (8–10, 17, 18). Regional evidence from the Gulf Cooperation Council (GCC) indicates that infant and neonatal mortality across the region is shaped by a comparable mix of sociodemographic, macroeconomic, and health-system determinants, and that neonatal sepsis and other quality-sensitive causes of death remain clinically important even in health systems with high overall coverage (4, 11). Saudi hospital-based studies have similarly reported that prematurity, low birth weight, and neonatal sepsis remain leading causes of NICU mortality, though such studies are facility-specific and cannot be generalized to national trends (4, 11).

Despite this body of work, few published analyses have examined recent (2018–2023) year-to-year NMR and U5MR dynamics in Saudi Arabia using a single standardized, internationally comparable data source spanning the Vision 2030 implementation period; most available Saudi-focused evidence is either facility-based, historical in scope, or drawn from non-harmonized national sources (4, 7). This is a modest, descriptive gap: it does not imply that national mortality levels are unknown, but that a focused, standardized annual description of this specific period has not been reported.

This study addresses that gap by describing annual NMR and U5MR trends in Saudi Arabia from 2018 to 2023 using WHO-GHO data. The objective is descriptive: to characterize the direction and approximate magnitude of year-to-year change in both indicators over this period, and to discuss these patterns against the regional and international literature. This study does not measure healthcare quality, patient safety, or COVID-19-specific disruption, and does not attempt to establish that observed trends were caused by any particular determinant.

## 2 Materials and methods

### 2.1 Study design

This is a descriptive ecological analysis of secondary, publicly available, population-level data. The unit of analysis is the national population of Saudi Arabia. No individual-level data, clinical records, or human subjects were involved. Given six annual data points per indicator, the design is intended to characterize the pattern of national mortality estimates over time, not to test causal hypotheses or to model time-series structure formally (e.g., autocorrelation, structural breaks, or interrupted time-series effects were not assessed and would require a longer series).

### 2.2 Data source and setting

Data were extracted from the WHO Global Health Observatory (WHO-GHO) database (8). WHO-GHO national child mortality estimates are produced by the UN Inter-agency Group for Child Mortality Estimation (UN IGME), which fits a statistical model to available civil-registration, census, and household-survey data for each country and generates estimates with associated uncertainty intervals (UI) to account for gaps and inconsistencies across underlying data sources. These are therefore modeled estimates rather than direct annual counts, and country-level data completeness and the underlying mix of data sources can vary by year. This study used the WHO-GHO point estimates; UI were not extracted as part of the dataset compiled for this analysis, which is noted as a limitation in Section 4.6. All estimates are expressed per 1,000 live births. The study period (2018–2023) was chosen to correspond to the implementation phase of the Saudi Vision 2030 healthcare reform agenda.

### 2.3 Outcome variables

Two variables were analyzed: (1) NMR, deaths within the first 28 completed days of life per 1,000 live births; and (2) U5MR, deaths between birth and exactly five years of age per 1,000 live births. Calendar year (2018–2023) was treated as the independent variable.

### 2.4 Ethical considerations

This study used exclusively publicly available, aggregated national-level secondary data from WHO-GHO. No individual patient data, personal identifiers, or confidential health records were accessed. Informed consent was not required. Formal institutional ethics approval was not required per applicable guidelines for secondary analysis of publicly available aggregate data.

### 2.5 Statistical analysis

Data were organized in Microsoft Excel and analyzed using SPSS version 27 and R (version 4.3). Two complementary approaches were used:

- Descriptive statistics and Annual Percentage Change (APC): Annual values, mean, minimum, maximum, and range were calculated for both indicators. Year-to-year APC was calculated as [(Yt − Yt − 1) / Yt − 1] × 100. This is the primary analytical approach, as it directly summarizes the observed data without additional modeling assumptions.
- Unadjusted linear regression: Mortality rate was modeled as a linear function of year to summarize the average annual rate of change (slope, *β*) over the period. With only six annual observations, this regression has very limited statistical power; consequently, *p*-values are reported alongside the slope for completeness only, and the presence or magnitude of a trend is described using the estimated slope and the annual pattern in Tables 1, 2 rather than relying on statistical significance. A non-parametric Mann–Kendall test and a separate “sensitivity analysis” step were considered but were not included as primary results, because with *n* = 6 both provide effectively the same directional information as the regression slope while adding limited independent information; where relevant, this cross-method consistency is noted narratively in Section 3.

**Table 1:** Neonatal mortality rate (NMR) in Saudi Arabia, 2018–2023 (source: WHO-GHO).

Year	NMR (per 1,000 live births)	Year-on-year change (%)
2018	3.6	—
2019	3.6	0.0%
2020	3.2	−11.1%
2021	3.1	−3.1%
2022	3.1	0.0%
2023	3.0	−3.2%

**Table 2:** Under-five mortality rate (U5MR) in Saudi Arabia, 2018–2023 (source: WHO-GHO).

Year	U5MR (per 1,000 live births)	Year-on-year change (%)
2018	6.1	—
2019	5.9	−3.3%
2020	5.7	−3.4%
2021	5.6	−1.8%
2022	6.4	+14.3%
2023	6.2	−3.1%

Given the small number of data points, all statistical results are interpreted descriptively; the absence of statistical significance does not imply the absence of a meaningful change, and conversely, an observed change should not be over-interpreted as confirming any particular explanation.

## 3 Results

### 3.1 Neonatal mortality rate (NMR), 2018–2023

Table 1 presents annual NMR values. NMR was stable at 3.6 per 1,000 live births in 2018–2019, declined to 3.2 in 2020, and declined further and more gradually to 3.0 by 2023. Over the full period, NMR fell by 0.6 per 1,000 live births (a 16.7% relative reduction), concentrated mainly in the 2019–2020 interval. The unadjusted linear slope was *β* = −0.12/year (*p* > 0.05).

### 3.2 Under-five mortality rate (U5MR), 2018–2023

Table 2 presents the U5MR trend. U5MR declined from 6.1 in 2018 to 5.6 in 2021, rose to 6.4 in 2022 (the largest single-year change in the dataset, +14.3%), and partially returned to 6.2 in 2023. The unadjusted linear slope over the full period was *β* = +0.03/year (*p* > 0.05), reflecting the offsetting effect of the early decline and the 2022 rise rather than the absence of any year-to-year change.

The 2022 rise and 2023 partial reversal are described here as an observed data pattern only; possible explanations, including the temporal coincidence with the post-acute phase of the COVID-19 pandemic, are considered in Section 4 as one hypothesis among others, since this study did not analyze COVID-19 incidence, service-utilization, or cause-specific mortality data.

### 3.3 Descriptive summary and regression results

Table 3 summarizes descriptive statistics, and Table 4 summarizes the unadjusted regression slopes. The narrow ranges (NMR: 0.6; U5MR: 0.8) indicate limited year-to-year volatility over the period as a whole, notwithstanding the single-year 2022 U5MR change. The NMR slope is directionally consistent with a gradual decline; the near-zero U5MR slope reflects the 2022 spike offsetting the earlier downward pattern rather than a genuinely flat trajectory throughout the period.

**Table 3:** Descriptive statistics for NMR and U5MR, Saudi Arabia, 2018–2023.

Indicator	Mean	Minimum	Maximum	Range
NMR	3.27	3.0	3.6	0.6
U5MR	5.98	5.6	6.4	0.8

**Table 4:** Unadjusted linear regression results for NMR and U5MR.

Indicator	β (slope)	Direction	*p*-value
NMR	−0.12	Decreasing	>0.05
U5MR	+0.03	Little net change	>0.05

## 4 Discussion

### 4.1 Principal findings

Between 2018 and 2023, NMR in Saudi Arabia declined gradually from 3.6 to 3.0 per 1,000 live births, and U5MR fluctuated within a narrow range around a mean of 5.98, with a transient rise in 2022. Both indicators remained low by international standards throughout the period. These are descriptive observations; this study did not measure healthcare quality, patient safety, or system performance directly, and the data cannot establish why the observed pattern occurred.

### 4.2 Comparison with regional and global evidence

These results extend the long-run description reported by Al Nafeesah (10), who documented a decline in Saudi Arabia’s U5MR from over 100 per 1,000 live births in the early 1970s to single-digit rates today; the present analysis adds annual granularity for the most recent, comparatively stable phase of that trajectory. Consistent with global patterns in which neonatal conditions represent a growing share of under-five mortality in low-mortality settings (2), NMR represented roughly half of U5MR throughout the study period in this dataset.

Regional GCC evidence provides useful context but does not permit a causal reading of the Saudi data. A systematic review of infant mortality determinants across GCC countries found that sociodemographic factors (e.g., fertility patterns, maternal employment and education), macroeconomic conditions, and health-system resources each contribute to variation in infant mortality across the region (4), underscoring that any interpretation of Saudi trends should consider this wider set of determinants rather than healthcare quality alone. Separately, a GCC-wide systematic review and meta-analysis of neonatal sepsis pathogens found that antimicrobial-resistant organisms remain a clinically significant contributor to neonatal morbidity and mortality across the region, including Saudi Arabia (11), which is consistent with—though does not directly confirm—the possibility that quality-sensitive causes of neonatal death remain relevant locally.

Regarding the 2022 U5MR rise, international bodies documented widespread disruption to essential maternal, newborn, and child health services during the COVID-19 pandemic, including deferred immunization and reduced care-seeking, in many countries during 2021–2022 (12). This is one plausible explanation for the pattern observed in the Saudi data, consistent with the timing of the rise and its partial reversal by 2023; however, because this study did not analyze COVID-19 incidence, service-utilization, or cause-specific mortality data for Saudi Arabia specifically, this remains a hypothesis rather than a demonstrated explanation, and alternative explanations (e.g., data revision, sampling variation in the underlying UN IGME model inputs) cannot be excluded.

### 4.3 Interpreting mortality trends in relation to healthcare quality

Mortality indicators are influenced by, but are not a direct measure of, healthcare quality or patient safety (13, 14). The literature on high-quality health systems suggests that as mortality approaches low absolute levels, further gains increasingly depend on the quality of perinatal and neonatal care rather than only on access (6, 13); this is a hypothesis supported by prior literature, and the present dataset—which does not include measures of clinical quality, patient safety events, or care processes—cannot itself test it. The observation that NMR constitutes a substantial and apparently stable share of U5MR in this dataset is consistent with, but does not prove, this broader pattern.

### 4.4 Policy considerations

These descriptive findings may be relevant to Saudi Arabia’s Vision 2030 health transformation agenda, which includes quality improvement and patient safety among its priorities, but the six-point national time series used here cannot support specific facility-level or clinical recommendations. Two considerations follow directly from the data: (1) continued standardized annual monitoring of NMR and U5MR would help distinguish genuine shifts (such as the 2022 U5MR change) from year-to-year variation; and (2) because this analysis cannot determine the cause of the 2022 fluctuation, linking national mortality surveillance to cause-specific and service-utilization data would help clarify whether such fluctuations reflect care disruption, data variation, or another factor. Broader recommendations regarding NICU capacity, workforce training, or specific clinical protocols would require direct clinical or facility-level data and are beyond what this ecological dataset can support.

### 4.5 Strengths and limitations

Strengths include the use of a standardized, internationally comparable, publicly available data source (WHO-GHO/UN IGME) and a focus on a policy-relevant period aligned with Vision 2030 implementation.

Limitations are substantial and should guide interpretation. First, the ecological design cannot support individual-level or causal inference. Second, with only six annual observations, statistical power is very limited; non-significant regression results do not imply the absence of change, and small fluctuations can appear as a “trend” or its absence depending on which years are included. Third, WHO-GHO/UN IGME estimates are statistically modeled from available civil-registration, survey, and census data rather than being direct annual counts, and this study did not extract or analyze the associated uncertainty intervals; future analyses should incorporate these intervals directly. Fourth, cause-specific mortality data (e.g., prematurity, sepsis, congenital anomalies, birth asphyxia) were not available in this dataset and could not be examined. Fifth, this study did not measure healthcare quality, patient safety, or COVID-19-specific service disruption; any discussion connecting the observed trends to these factors in Section 4 is offered as a hypothesis for future research, not a finding. Sixth, potential confounders—socioeconomic change, subnational disparities, healthcare workforce ratios, and specific policy interventions—were not controlled for. Finally, generalizability is limited to the Saudi national level; comparative subnational and GCC regional analyses using harmonized data would add useful context.

## 5 Conclusion

Between 2018 and 2023, NMR in Saudi Arabia declined gradually from 3.6 to 3.0 per 1,000 live births, and U5MR fluctuated within a narrow range, including a transient rise in 2022 that had largely reversed by 2023. Both indicators remained low by international comparison throughout the period. These are descriptive, population-level observations drawn from six annual WHO-GHO estimates; the dataset does not include measures of healthcare quality, patient safety, or COVID-19-specific disruption, and interpretations connecting the observed patterns to these factors should be treated as hypotheses for future research rather than conclusions of this study. Continued standardized monitoring, together with future work incorporating cause-specific mortality, subnational data, WHO/UN IGME uncertainty intervals, and direct quality-of-care indicators, would allow firmer conclusions about the drivers of recent mortality patterns in Saudi Arabia and their relevance to SDG 3.2 and Vision 2030.

## Data Availability

Publicly available datasets were analyzed in this study. This data can be found at: https://www.who.int/data/gho.
